# Overview of Canada's national dementia research consortium CCNA

**DOI:** 10.1002/alz70855_102886

**Published:** 2025-12-29

**Authors:** Howard Chertkow

**Affiliations:** ^1^ Baycrest Academy for Research and Education, Toronto, ON, Canada

## Abstract

**Background:**

It is not clear how best to coordinate, facilitate, and catalyze dementia research at the national level of most countries.

**Method:**

Literature search on CCNA and qualitative review of website of CCNA, newsletters, and grant renewals in Canada.

**Result:**

The Canadian Consortium on Neurodegeneration in Aging (CCNA) was created by the Canadian federal government in 2014 through the Canadian Institutes for Health Research (CIHR). Two five‐year funding cycles have occurred following peer review, and a third cycle (Phase 3) has just begun. Twenty national teams were established, with research topics focussing on national research strengths, spanning from basic to clinical science to health resource systems. Teams have facilitated greater interaction. Responding to the needs of researchers within the CCNA teams, a unique sample of 1,173 deeply phenotyped patients with various forms of dementia was accrued and studied over eight years (COMPASS‐ND). In the second phase of funding (2019–2024), a national dementia prevention research program (CAN‐THUMBS UP) was set up. In a short time, this prevention program became a member of the World Wide FINGERS prevention consortium. Cross‐cutting programs were established to support the enterprise, focussing on KT, Training, and Sex and Gender in Dementia. A unique group integrated persons with lived experience into the national research program (EPLED, Engagement of People with Lived Experience of Dementia). An emphasis was placed on developing knowledge and capacity and procedures for investigating dementia in the Canadian Indigenous communities, where it is higher than other populations.

Objective measures have demonstrated increased synergy and productivity among Canadian dementia researchers since establishment of CCNA, along with leveraging of new grants equal to the CIHR funding. More than 600 journal articles have resulted from CCNA work, with higher impact than corresponding non‐CCNA work. The network has had demonstrable impact on policy‐makers and been a conduit towards greater impact of the research community nationally and internationally.

**Conclusion:**

Enhancement of synergy and networking have contributed to the considerable success of CCNA by all measures. CCNA is evidence that an organized “centrally‐organized” approach to dementia research can catalyze important progress nationally and yield significant and measurable results.

